# A working model for cytoplasmic assembly of H/ACA snoRNPs

**DOI:** 10.1002/1873-3468.70154

**Published:** 2025-08-29

**Authors:** Alberto Angrisani, Maria Furia

**Affiliations:** ^1^ Department of Biology University of Naples “Federico II”, Complesso Universitario Monte Santangelo Italy

**Keywords:** dyskeratosis, dyskerin, pseudouridylation, ribosomopathies, snoRNPs biogenesis, translational regulation

## Abstract

Pseudouridylation occurs on all types of cellular RNAs. As a catalytic component of nuclear H/ACA ribonucleoproteins (RNPs), the pseudouridine synthase dyskerin exerts a general impact on multiple fundamental cellular processes. Although this protein has been investigated in detail, its cytoplasmic roles have been largely overlooked, despite the identification of a minor splice variant showing a prevalent cytoplasmic localization. In this perspective, we discuss the role of this minor isoform in the composition, assembly, and function of H/ACA RNPs. On this basis, we propose a model in which cytoplasmic regulation modulates the integration of distinct dyskerin isoforms within preassembly complexes, consequently also influencing their nuclear import. This mechanism could govern pseudouridylation, cellular growth, and mRNA translation.

**Impact statement**

Human Dyskerin is the pseudouridine‐synthase component of H/ACA RNPs. Two isoforms have been characterized: the abundant Iso1, mainly nuclear, and Iso3, mainly cytoplasmic but occasionally imported into nuclei. We propose a model accounting for a regulated participation of both isoforms in the cytoplasmic pre‐assembly of H/ACA RNPs.

## Graphical abstract

Dyskerin is the component of nuclear H/ACA ribonucleoproteins (RNPs) endowed with pseudouridine synthase catalytic activity. Two isoforms of human dyskerin have been characterized: the abundant Iso1, mainly nuclear, and the shorter Iso3, mainly cytoplasmic but occasionally imported into nuclei. In this perspective, we propose a model accounting for a regulated participation of both isoforms in the cytoplasmic preassembly of H/ACA RNPs.

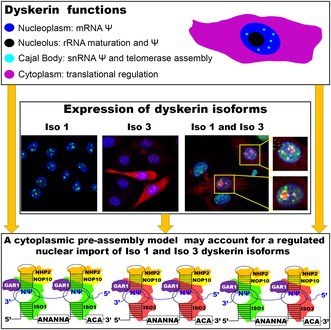

## Abbreviations

**AS**, alternative splicing

**CBs**, Cajal bodies

**C‐Ter**, carboxy terminal

**dbEST**, expressed sequence tags database

**DKCX**, X‐linked dyskeratosis congenita

**ER**, endoplamic reticulum

**ES**, embryonal stem

**HHS**, Hoyeraal–Hreidarsson syndrome

**hTERC**, human telomerase RNA component

**hTERT**, human telomerase reverse transcriptase

**IDRs**, intrinsically disordered regions

**Iso1**, dyskerin Isoform 1

**Iso3**, dyskerin Isoform 3

**ncRNAs**, noncoding RNAs

**NLS**, nuclear localization signal

**NS**, nuclear speckles

**N‐Ter**, NH2‐terminal

**P‐bodies**, processing bodies

**PUA**, pseudouridine synthase and archaeosine transglycosylase

**RNPs**, ribonucleoparticles/ribonucleoproteins

**scaRNAs**, Cajal body‐specific RNAs

**SG**, stress granules

**snoRNAs**, small nucleolar RNA

**snoRNPs**, small nucleolar RNPs

**TruB**, tRNA pseudouridine synthase B

**UTR**, untranslated terminal region

**Ψ**, pseudouridine

Pseudouridylation is the most abundant post‐transcriptional modification of cellular RNAs and exerts wide effects on gene expression [1]. Nowadays, the considerable potential for RNA pseudouridylation to affect critical cellular functions is continuously highlighted by results of quantitative assays profiling [2]. New techniques demonstrate the high prevalence of this RNA modification, and many reports continuously expand the repertoire of its crucial roles in cellular activities. In fact, pseudouridylation can modulate RNA stability and folding and occurs on all RNA types [1, 2, 3]. This widespread RNA modification is known to have a direct impact on ribosome biogenesis [4], translation initiation, fidelity, and regulation [5, 6, 7], and can also modulate splicing [8, 9].

**Fig. 1:**
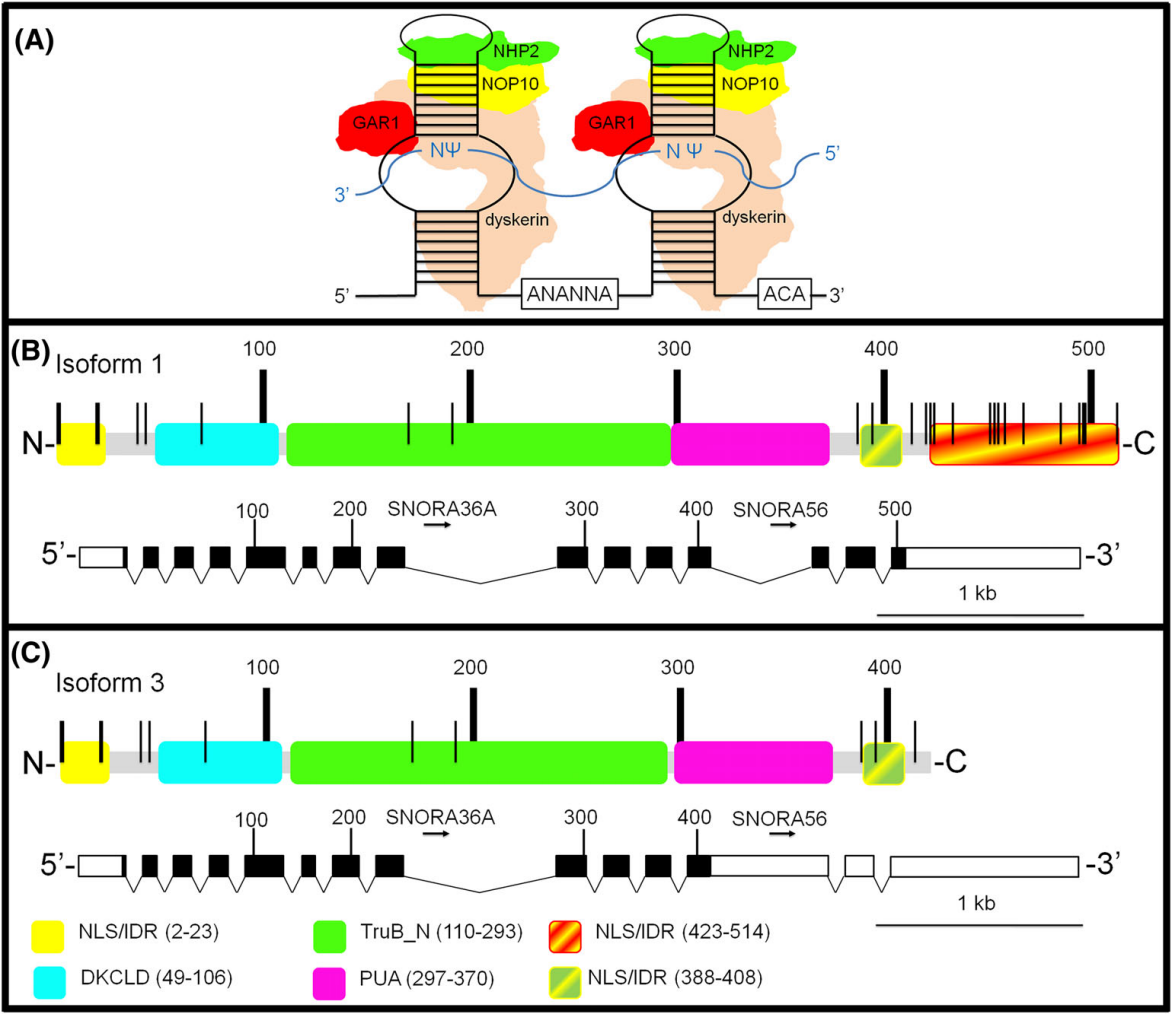
Structure of dyskerin‐containing H/ACA snoRNPs. (A) Organization of H/ACA snoRNPs. H/ACA snoRNA backbone (black) and target RNA (blue). N represents any nucleotide, Ψ the modified uridines. Proteins composing the heterotetrameric complex are depicted (GAR1, NHP2, NOP10, and dyskerin). (B, C) On the top, diagrams representing the structure of dyskerin Iso1 (B) and Iso3 (C) proteins. Functional domains are retrieved from the Pfam database; the role of the conserved DKCLD region remains elusive. Positions of the NLS/IDR region are according to [20]. Numbers mark the amino acid position according to Iso1; vertical rods the site of post‐transcriptional modifications listed in (Table 1). Below, organization of Iso1 (B) and Iso3 (C) mRNAs. Note that only exons and introns 8 and 12, including SNORA36A and SNORA56, are depicted to scale.

Human dyskerin (UniProt # O60832) [10], encoded by the *DKC1* gene, belongs to a highly evolutionarily conserved family of RNA‐guided pseudouridine synthases, whose members are essential for life from archaea to humans (reviewed in [11]). Dyskerin acts as a catalytic player in the pseudouridylation process, in concert with a core set of three highly conserved proteins (NOP10, NHP2, and GAR1) and a small RNA carrying the typical H/ACA box signature [12, 13]. H/ACA RNAs share a characteristic hairpin‐hinge‐hairpin tail secondary structure, where each hairpin is associated with a protein heterotetrameric core. A single‐stranded tract that connects the two hairpins includes the conserved H box, while the tail encloses the conserved ACA box, located three nucleotides upstream from the 3′ terminus [14] (Fig. 1A). Additional proteins, such as the assembly factors SHQ1 and NAF1, are only transiently associated at the initial steps of the complex assembly [15]. Unlike eukaryotic stand‐alone pseudouridine synthases (PUS enzymes), the specific small RNA that is engaged in the assembly of the active H/ACA RNPs defines the biological role of these complexes. The association with H/ACA small nucleolar RNAs (snoRNAs) mainly controls ribosomal RNA (rRNA) pseudouridylation, which takes place in the nucleoli [16]. In contrast, the association with Cajal body‐specific RNAs (scaRNAs) at Cajal bodies (CBs) in the nucleus allows the formation of the small Cajal body‐specific ribonucleoproteins (scaRNPs), specifically dedicated to the modification of spliceosomal snRNAs [17]. At CBs, the interaction with human telomerase RNA component (hTERC), which harbors an H/ACA domain, allows dyskerin to also participate in the assembly of the telomerase holoenzyme [18, 19].

The more abundant isoform of dyskerin, hereafter referred to as Iso1, contains two nuclear localization signals (NLSs) situated at the N‐terminal (N‐Ter) and C‐terminal (C‐Ter) ends, respectively. These signals facilitate strong nuclear localization, predominantly in nucleoli and CBs. The functional domains of the protein include a Pseudouridine synthase and Archaeosine transglycosylase (PUA) domain, which is responsible for RNA recognition, tRNA pseudouridine synthase B (TruB) motifs, which play a catalytic role in pseudouridylation [20], and various intrinsically disordered regions (IDRs) that can mediate the assembly with different interacting partners [11, 21, 22]. Notably, human *DKC1*, like its Drosophila orthologue [23, 24], has a coding‐noncoding organization. SNORA36A and SNORA56, two rRNAs targeting H/ACA snoRNAs [25], are in fact embedded within introns 8 and 12, respectively [26] (Fig. 1B,C). As shown in Fig. 1C, *DKC1* also encodes a minor truncated dyskerin splice variant, hereafter referred to as Iso3, that lacks the C‐Ter NLS while retaining all other functional domains. Unsurprisingly, this isoform shows a predominant cytoplasmic localization [26]. Given that the generation of Iso3 by alternative splicing (AS) implies the retention of the whole intron 12, it is reasonable to assume that this event interferes with the biogenesis of SNORA56, which is reported to act as a microRNA precursor [27] and to promote several types of tumors [28, 29]. Finally, multiple sites of protein modification, mainly phosphorylation and sumoylation, have been mapped within the dyskerin protein sequence (Table 1). The accumulation of modification sites at the Iso1 C‐Ter region, which is missing in Iso3, represents an additional distinctive trait.

**Table 1:** List of the post‐transcriptional modifications (PTMs) demonstrated in dyskerin, as derived from Uniprot data [10].

Shared by Isoform 1 and 3		Isoform 1‐specific	
Position	PTM	Position	PTM
1	Initiator methionine elimination	420	Phosphoserine (note 420 is Arg in Isoform 3)
2	*N*‐Acetylalanine	422	Phosphoserine
20	SUMO2	424	SUMO2
21	Phosphoserine	433	SUMO2
39	SUMO2	451	Phosphoserine
43	SUMO2	453	Phosphoserine
70	Phosphothreonine	455	Phosphoserine
170	Phosphoserine	458	Phosphothreonine
191	SUMO2	467	SUMO2
387	Phosphoserine	485	Phosphoserine
394	SUMO2	494	Phosphoserine
413	SUMO1 and 2	496	Phosphothreonine
		497	Phosphothreonine
		513	Phosphoserine

Human dyskerin is widely recognized as a multifunctional protein involved in several aspects of cell homeostasis, including cell proliferation, ribosome biogenesis, and telomere stability [30]. However, the protein has additional moonlight roles in vesicular trafficking [31], energy metabolism [32], stress/DNA damage response [33, 34, 35, 36], and mRNA splicing and translatability [8, 9]. Dyskerin is also relevant in germ cell stemness [37, 38], somatic cell niches [39], and differentiation [40]. Stem or progenitor cells require elevated levels of both telomerase activity and ribosome biogenesis to support growth and proliferation, making dyskerin malfunction disadvantageous in this context. This is supported by the poor reprogramming efficiency and the inability of induced pluripotent stem cells (iPSCs) from fibroblasts of X‐linked dyskeratosis congenita (DKCX) patients, who carry loss‐of‐function mutations in *DKC1*, to retain their undifferentiated state [37]. In addition, a more direct link was revealed by Fong *et al*. [38], who showed that dyskerin‐participated RNPs act as transcriptional coactivators for OCT4/SOX2, regulating Nanog gene expression in mammalian embryonal stem (ES) cells. Since dyskerin stabilizes H/ACA snoRNAs [41], faulty protein expression may also affect the quantity or localization of these small RNAs and their derivatives, whose levels are known to be critical for differentiation. Many families of H/ACA snoRNAs have been shown to be differentially expressed in mouse ES cells, supporting the hypothesis that changes in their levels may cause differentiation [42]. In accordance, depletion of Drosophila pseudouridine synthase affects tissue regeneration [43], maintenance of somatic stem cell niches [39], as well as growth, proliferation, and differentiation of somatic cells [40]. Drosophila pseudouridine synthase is also required for the preservation of the male germline stem cell lineage [44] and oocyte specification [45]. Finally, the H/ACA RNP complex is also actively involved in the modification of many families of germline small RNAs in plants [46], further reinforcing dyskerin's critical role in stemness.

Dyskerin can thus intersect many cellular pathways and, although most of the underlying molecular mechanisms remain to be fully elucidated, its relevance is confirmed by the observation that loss‐of‐function causes two congenital ribosomapathies: DKCX (OMIM: #305000) [47] and Hoyeraal–Hreidarsson syndrome (HHS) [20, 48]. Both diseases show a distinctive triad of nail dystrophy, mucosal leukoplakia, and reticular hyperpigmentation, alongside bone marrow failure, stem cell defects, mental impairment, premature aging, and tumor susceptibility [48]. Aplastic anemia from bone marrow failure often precedes DKCX diagnosis [48]. Conversely, overexpression of dyskerin is linked to various sporadic cancers and serves as a marker for poor prognosis, indicating the importance of precise dyskerin expression control (reviewed in [11]).

As mentioned above, dyskerin expression may influence the level or localization of H/ACA snoRNAs [41], which can be processed into microRNAs [49] or smaller RNAs that regulate AS [50, 51]. All these small RNAs can assume extra‐nuclear localizations, including exosomes or extracellular fluids [52], and are often implicated in diverse biological and pathological processes [53]. As recently described, H/ACA snoRNAs can also play uncanonical roles, independently from their target specificity, in either nuclei or cytoplasm [8, 9, 54, 55]. A striking example is SNORA73, which targets mRNAs of secreted proteins, forming a ternary complex with 7SL RNA that aids in protein secretion across the ER [55]. Depleting the H/ACA RNP core proteins reduces SNORA73 levels and secretion, highlighting their role in SNORA73 biogenesis, nuclear export, and ER protein translocation [55]. As noticed by the authors, Iso1 was confined into the nuclei, while Iso3 was enriched in the ribosome fraction, leading to the conclusion that ‘the cytosolic isoform of *DKC1* is more likely directly involved in protein translocation through the pathway’ [55]. In this perspective, we propose a model considering the specific localization of dyskerin isoforms, which modulates the integration of distinct dyskerin isoforms within H/ACA RNP preassembly complexes, consequently also influencing their nuclear import and subsequently pseudouridylation, cellular growth and mRNA translation.

## Dyskerin: in and out of the nucleus

The cytoplasmic trafficking of dyskerin has not been thoroughly investigated, likely due to its high nuclear concentration which obscures direct visualization of a lower‐level presence in the cytoplasm. Consequently, a primary hint about the cytoplasmic roles of dyskerin was limited to an unrelated study that reported its association with the S19 ribosomal protein [56]. The first direct insight into dyskerin's cytoplasmic activities was reported in 2011, when we identified the truncated splice variant Iso3, whose distinctive trait was the full retention of intron 12 [26]. Notably, a *DKC1* G→A splice‐site mutation at IVS12 + 1 disrupting the intron 12 splice donor site was previously reported in a male infant with HHS, who died at age 2 [57]. Intron retention was also observed in a Chinese family exhibiting DKCX pathogenic symptoms [58]; more recently, a *de novo DKC1* splice‐site variant in a patient presenting HHS clinical features has also been described [59]. Indeed, considering the dbEST database [60] and a previous report [61], alternative splicing appears to occur frequently in the processing of dyskerin pre‐mRNA. It is plausible that it might represent an autoregulatory mechanism modulating the generation of distinct functional products across different cell types [61].

## Iso3: a new player in the RNA‐guided pseudouridylation process

The dyskerin Iso3 variant is expressed at a low level and is less stable than Iso1, being tightly regulated at a post‐transcriptional level by proteasome‐mediated degradation [36]. The multiple sites of protein modifications that are located in the absent Iso1 C‐Ter region (see Fig. 1B,C and Table 1) might contribute to its reduced half‐life. Notably, in a large‐scale analysis, the intracellular localization of Iso3 varied greatly, likely due to cell metabolic conditions. In fact, Iso3 is detectable in the cytoplasm of many cells, whereas it can be observed in the nucleus, or in both cell compartments, in others [22, 36]. Within nuclei, where it is presumably protected from prompt proteolytic degradation, Iso3 concentrates in nucleoli and CBs, which are two closely connected organelles and the primary localization of full‐length Iso1. Hence, the lack of the C‐Ter NLS proved to be irrelevant not only for nuclear but even for proper subnuclear localization of the protein. This is in good agreement with a previous study that indicated that the N‐Ter and the C‐Ter NLSs of Iso1 were both sufficient, although at a lower efficiency, for nuclear localization, whereas together they acted synergistically [62]. In accordance with the retention of both PUA and TRuB motifs, a recent report confirmed that Iso3 is endowed with pseudouridylation activity [63]. In fact, as tested in a premature stop codon readthrough assay, the catalytic performance of this isoform was found to be markedly higher than that of Iso1 [63, 64]. Moreover, endogenous Iso3 has lately been found to associate with ribosomes, leading to the suggestion that it may regulate protein synthesis by mRNAs pseudouridylation [55]. Although it is plausible that the nuclear import of this isoform would occur under specific, perhaps tightly regulated, conditions [36], the notion that *DKC1* can generate alternatively spliced isoforms with different expression patterns, and perhaps distinct biological roles, needs to be taken into account. Even though Iso3 has been largely unnoticed for a long time, we and others connected its activity to lipid metabolism [65], redox response [32, 33, 34, 35, 36], and ribosome functionality [8, 9, 26, 36, 55, 63, 64]. Thus, in accordance with our previous data and more recent reports [8, 9, 55, 63, 64], we propose a model for H/ACA snoRNP assembly that includes Iso3 as a new player in the repertoire of dyskerin functions.

## A cytoplasmic assembly regulation working model of H/ACA snoRNPs

Assembly of H/ACA snoRNPs is a multistep process that entails multiple intermediate complexes assisted by transient interactions with different chaperones. According to a previous model [66], the first step starts with the precocious association of dyskerin with the assembly factor SHQ1, which interacts directly with the RNA‐binding domain of dyskerin in the cytoplasm, protecting it from binding spurious RNAs and impeding illicit aggregation or degradation [15]. Association of a dyskerin‐NHP2‐NOP10 heterotrimer with nascent H/ACA snoRNAs was then thought to be mediated by a fourth protein, NAF1, which is transiently recruited in the nucleus at the site of snoRNA transcription [15]. Nascent snoRNPs then move to CBs, where NAF1 is replaced by GAR1 and the mature complex is assembled. Active H/ACA snoRNPs finally move to nucleoli, where they are mainly involved in the processing and modification of rRNA while, as mentioned above, association with scaRNPs and assembly with hTERC would occur at CBs. Recently, several features of this traditional model have been questioned and refined. In fact, a more recent study based on quantitative SILAC proteomic analyses of purified complexes containing GAR1, NHP2, SHQ1, and NAF1 [67] identified new intermediate complexes and proposed to revise some points. The most relevant issue to the previously proposed assembly model is the finding that a preassembly complex containing dyskerin, SHQ1, NAF1, NOP10, and NHP2 could be formed independently of the presence of snoRNAs. This would imply that this step might occur in either the cytoplasm or nucleus. In accordance with this novel finding, we now propose an updated working model for cytoplasmic H/ACA snoRNP assembly that includes Iso3 as a new player in the highly regulated assembly process.

**Fig. 2:**
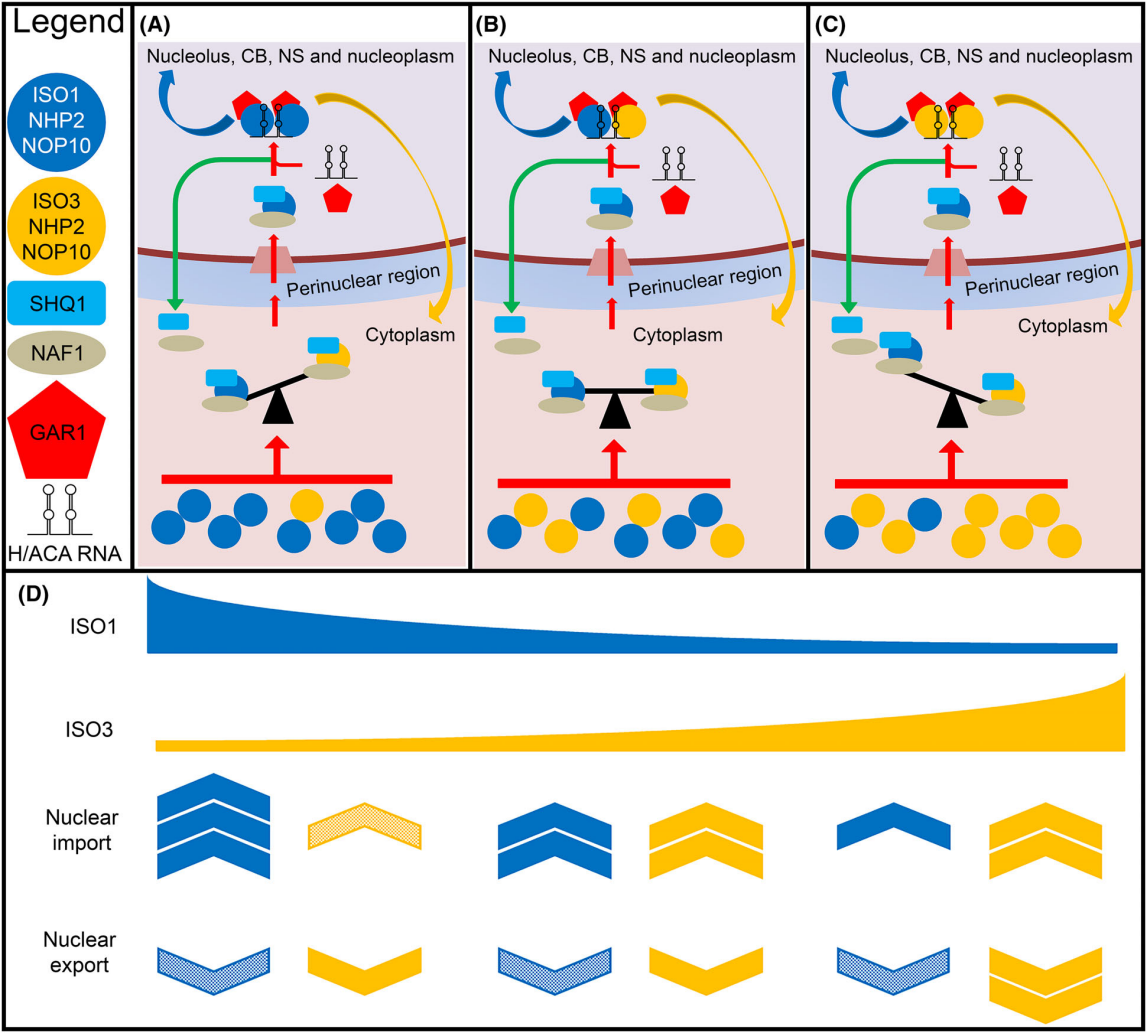
Cytoplasmic regulation assembly (CAR) model for H/ACA RNP biogenesis. As depicted in the legend, dyskerin‐NOP10‐NHP2 trimers are represented by circles, blue: containing Iso1, yellow: containing Iso3 in H/ACA RNP assembly. Binding of SHQ1 and NAF1 shuttling factors to the preassembly complexes takes place in the cytoplasm. The preassembly complexes, once gathered, are imported into the nucleus where the detachment of SHQ1 and the substitution of NAF1 by GAR1 and the binding to the specific small ncRNA (snoRNA/scaRNA/hTERC) occurs. Subsequently, each type of H/ACA mature complex concentrates into the subcellular structures where its specific function is required. (A) Due to the high cytoplasmic concentration of *neosynthesized* or *favored* Iso1, in most cells, the Iso1/Iso1 complex is preferentially assembled into nuclei where it is stably retained. (B) At intermediate levels of Iso1 and Iso3 cytoplasmic concentration, mixed complexes containing both isoforms are also assembled. It is reasonable to predict that this type of complex might favor Iso3's nuclear retention. Once in the nuclei, Iso3 could participate in the assembly of preribosomal particles, chaperone them to the cytoplasm, and eventually be retained in mature ribosomes [8, 55]. (C) Upon specific stimuli, or a variety of regulatory mechanisms, the Iso1/Iso3 cytoplasmic concentration ratio can be inverted, allowing the formation of Iso3/Iso3 alternative complexes. Iso3 is supposed to be involved in the assembly of preribosomal particles and should be exported to the cytoplasm with them, where it is in part retained in mature ribosomes [8, 55]. (D) The formation of the (A–C) complexes is correlated with Iso1 and Iso3 protein levels. The hypothesized nuclear import/export fluxes of the isoforms are depicted by the number and the intensity of arrows; pixeled arrows indicate minimal amounts.

In this cytoplasmic assembly regulation (CAR) model, we surmise that Iso3, although being prominently cytoplasmic, is generally outcompeted by Iso1 in a cytoplasmic preassembly process of H/ACA RNPs (Fig. 2A). The low abundance and instability of this isoform, and perhaps other mechanisms such as cytoplasmic subcompartmentalization, might contribute to its poor recruitment. Conversely, the catalytic activity of Iso3 and its ability to localize also into nucleoli and CBs [36] suggest its participation in the assembly of H/ACA snoRNPs. Thus, considering that overall cellular concentrations of Iso1 constantly largely exceed those of Iso3, we reason that this less abundant variant could outcompete Iso1 in the preassembly complexes only in the cytoplasm, and only under selected conditions.

According to our proposed model, the key parameter determining which of the two dyskerin isoforms will be recruited in the preassembly complex is the ratio of cytoplasmic *free* Iso3 levels and cytoplasmic ‘neosynthesized’ or ‘unfavored’ Iso1 levels. Hence, a crucial point of the model is the identification of the yet‐to‐be‐determined mechanisms, which would allow Iso3 to outcompete Iso1 and to eventually be transferred into the nuclei, where it likely escapes prompt degradation. To this end, several options seem plausible. The simplest and more direct possibility would be an increase in the efficiency of Iso3‐generating AS, which would simultaneously boost Iso3 synthesis and reduce Iso1 production, as shown in Fig. 2B,C. However, the same scenario might arise through a variety of pathways able to increase Iso3 levels or, alternatively, downregulate Iso1, through either transcriptional or post‐transcriptional mechanisms. The latter could include protein modifications, protein–protein or protein–RNA interactions, as well as microRNA‐regulated synthesis. Concerning the less‐characterized Iso3, enhanced chaperoning mechanisms, protective sequestration into cytoplasmic SG or P‐bodies [36] or other cytoplasmic structures can be envisioned. For Iso1, more detailed examples may be evoked, for instance a reduction or absence of the diverse post‐transcriptional modifications at the Iso1 C‐Ter region, which are believed to stabilize Iso1. Inappropriate aggregation or lack of protective interactions with proteins or RNAs might also influence Iso1 stability. Indeed, several cases of various ncRNAs, including long ncRNAs, interacting with dyskerin and influencing its stability, have been reported [68, 69, 70]. Finally, the importance of the 3′ UTR of Iso1 mRNA is exemplified by a family with DKCX carrying a deletion in this region [71]. Although the precise mechanisms remain to be defined, each of the diverse processes outlined above could potentially ‘unfavor’ Iso1 in the competition for the preassembly process. Notably, Iso3 nuclear import does not exclude the stable retention of Iso1‐containing nuclear complexes, as was observed [22, 36]; consequently, the overall cellular abundance of Iso1 would generally remain at a high level. The model proposed here represents a refined update, supported by a recent study [67] where we first revealed that H/ACA RNPs assembly may be regulated at the cytoplasmic level [11].

In summary, the model presented here is consistent with (a) the remark that Iso3 retains all sequences required for the interaction with the SHQ1 assembly factor [15] and (b) our previous results reporting that in some cells Iso1 was even displaced by Iso3 from inner nucleolar regions, while Iso1 levels in the dense fibrillar section and CBs may remain unvaried [22]. Considering that each H/ACA RNA contains two hairpins, our model also predicts that, depending on the relative cytoplasmic ratio of Iso1 to Iso3, complexes composed of either Iso1/Iso3 or Iso3/Iso3 might also be formed (Fig. 2B,C). Both types of complexes could import Iso3 into the nucleus, although to a different extent. Note that, in principle, each type of complex might also allow the export of preribosomal particles and/or H/ACA snoRNAs, or their derived RNA molecules, from the nucleus. Concerning Iso3, in previous works we already hypothesized that ‘this protein might directly interact with a subset of cytoplasmic mRNAs and/or with ribosomes, this way influencing translation’ [26] and ‘it might act as nuclear‐export chaperone for ribosomal particles’ [36] and/or ‘export of H/ACA RNAs’ [22, 36]. All these premises have been substantially supported by recent literature, given that Iso3 has been reported to associate with mature ribosomal structures [55] and to be potentially able ‘to control protein synthesis through pseudouridylation [8]’.

## Discussion

Here, we propose the CAR model that links old and recent data on the activity of the dyskerin splice variant Iso3 with the most recent update about H/ACA RNPs assembly [67].

Indeed, our proposed cytoplasmic preassembly model is not only compatible with novel findings, but also with the observation that, comparably to SHQ1, the three other components of the core heterotetramer (NHP2, NOP10, GAR1) also have a dual nucleo‐cytoplasmic localization. In addition, they coordinately interact with each other in the cytoplasm during the stress response, where they are co‐recruited into the stress granules (SGs) [36].

Nowadays, the finding that *DKC1* can generate alternatively spliced isoforms with perhaps distinct biological roles needs to be taken into account. For a long time, the predominant full‐length Iso1 has been assumed to be the only isoform active in the pseudouridylation process, with many additional roles routinely attributed to it, while no attempt to discriminate between alternative isoforms has been even pursued. Considering Iso3's relevance in the pseudouridylation process is now firmly established [63, 64], the most intriguing questions should concern the mechanism by which different dyskerin isoforms could be assembled into active RNPs, how their nuclear import is regulated, and whether they can exert different functions. In fact, many other proteins with specific nuclear functions also have additional subcellular localizations and perform distinct roles at those sites.

## Future perspectives

The main questions that presently arise are as follows: (a) Could the recruitment of different dyskerin isoforms confer distinct specificities to H/ACA modification complexes? (b) Could the nuclear capture of Iso3 solely downregulate its so far only partially understood cytoplasmic functions? (c) Does Iso3 play a role in the assembly of the active holo telomerase?

Concerning the first question, continuously growing evidence supports the direct involvement of Iso3 in the regulation of mRNA translatability and testifies its ability to perform pseudouridylation in the cytoplasmic context [63, 64]. Iso3's dual nucleo‐cytoplasmic localization also raises the possibility that this isoform could have a primary role in transducing nucleo‐cytoplasmic intracellular signals, and/or in the nuclear export of preribosomal particles or snoRNAs and derived small RNAs, as we first suggested [22, 26, 36]. The notion that the half‐life of Iso3 is strictly controlled by proteasome‐mediated degradation [36] is also consistent with the possibility of specialized functions, in accordance with data indicating that the exact stoichiometry of the four core proteins is tightly regulated and is required for the integrity of H/ACA RNPs [72]. In this regard, a promising hypothesis is that Iso3 may exhibit distinct binding affinities for different types of nuclear ncRNAs, including H/ACA RNAs, or for nuclear RNPs. Accordingly, this would allow Iso3 to assume preferential subnuclear localizations where it could exert distinct functions.

Until these aspects are clarified, the answers to the first and second questions remain undefined, since neither active participation in the pseudouridylation process nor any other role has been conclusively assessed for Iso3 in the nuclear context so far.

Regarding the third question, a positive hint derives from a study of MacNeil *et al*. [73], which found the interaction of the N‐Ter dyskerin region with hTERC to be crucial for preventing unchecked hTERC degradation [73]. More recently, the cryo‐EM structure of human telomerase with bound telomeric DNA confirmed that this dyskerin region interacts with hTERC terminal residues [74]. Given that these sequences are retained in Iso3, it is plausible that this isoform might participate in this crucial step of telomerase holoenzyme assembly. Thus, provided that all other necessary intermolecular interactions are maintained, the functionality of the resulting telomerase holoenzyme cannot be excluded. It is noteworthy that hTERT has a supplementary mitochondrial localization, enhancing respiration and protecting cells against oxidative stress and apoptosis (reviewed in [75]). The observation that Iso3 also improves mitochondrial functionality [32] could suggest a potential functional relationship between these proteins. However, further investigation is warranted to determine whether this isoform, due to its predominant cytoplasmic localization, can transmit a nucleus‐mitochondria signal, interact with hTERT in the cytoplasm, or even within the mitochondria.

In conclusion, since new nuclear and cytoplasmic functions are continuously evoked for dyskerin, identifying the specific isoforms involved in various nuclear and cytoplasmic functions, as well as their interactions, should be a priority for future research. These insights will deepen our understanding of the molecular basis of DKCX and HHS.

## Author contributions

AA contributed to the conceptualization and design of the manuscript, database analyses, and feedback on the final version of the manuscript. MF contributed to the conceptualization and design of the manuscript, writing of the original draft and the final manuscript, supervision, and feedback on the complete final version.
